# Persistent postsurgical pain—How can we make progress?

**DOI:** 10.1080/24740527.2019.1627849

**Published:** 2019-07-30

**Authors:** Henrik Kehlet

**Affiliations:** Section of Surgical Pathophysiology, Rigshospitalet, Copenhagen University, Copenhagen, Denmark

The current issue of the *Canadian Journal of Pain* contains a number of articles from a recent annual symposium from the Michael G. DeGroote Institute for Pain Research and Care. The main theme of the Institute has been “Persistent Postsurgical Pain—A Model for the Study of Chronic Pain,” and the annual symposia summarize recent topics of interest within the main theme and present research results supported by the Institute.

It is now more than 20 years since persistent postsurgical pain was determined to be an important clinical problem, and we have seen a virtual flooding of publications, including many recent reviews.^1–6^ However, despite the topic being a worldwide focus, we have to admit that real progress to decrease the problem has been limited, largely because of the multiple pathogenic mechanisms. It is therefore laudable that the DeGroote Pain Institute continues the multidisciplinary effort and collaboration to maintain focus on the specific role of the pre-, intra-, and postoperative pathogenic factors. The current special issue contains articles that address chronic postsurgical pain from the perspective of risk factors, measurement, and assessment, as well as mechanisms and treatment aspects.

Concerning the two papers on risk factors, Quinlan^7^ discusses the early and late post-caesarean delivery pain problems (about 10%) with an emphasis on the interaction between depression, sleep disturbances, and the mother’s quality of life, important topics for which more research is required due to the large number of operations. However, in addition to these risk factors and sequelae, more specific research is required on surgical technique and risk of nerve injury. The paper by Ashoorion et al.^8^ outlines their protocol for a systematic review on predictors of persistent pain after total knee arthroplasty, another a significant clinical problem with little progress despite many descriptive and randomized controlled analgesic trials. Due to the multiple pathogenic factors, the review results are awaited with interest, hopefully leading to improved strategies for prevention or treatment.

Regarding the issue of measurement and assessment, the paper by Khan et al.^9^ is important because it suggests that pain data collection be performed electronically vs. the traditional paper-and-pencil approach. The authors recommend that the positive data from a small group of breast cancer patients should be followed up by larger samples in other procedures. The paper by Ouellette et al.^10^ brings up an important commentary on the barriers for optimized pain assessment and treatment using the “SMArTVIEW” intervention, which is a three-component intervention with automatic monitoring in hospitals, home care, and support as well as self-management education based on specific cardiovascular surgery data. The proposed approach may have major implications for clinical research addressing barriers to effective postoperative pain management and should serve as a stimulus to be introduced in other surgical procedures with potential different barriers. The paper by Gilron et al.^11^ emphasizes that assessment of postoperative pain must be optimized regarding procedure- and patient-specific assessment and at rest and during well-defined procedure-relevant function. In addition, these measures must be related to opioid requirements and specific functional outcomes. Although representing an easily understandable and important message, most recent perioperative pain trials still have suboptimal pain assessments and limited information on its functional consequences.

In an article addressing potential mechanisms of chronic postsurgical pain, Linher-Melville and Singh^12^ raise an important question based on their established experimental model to further evaluate sex differences in pain perception. The potential role of cannabinoid treatment is discussed in this paper, representing an important topic given the more widespread use of these agents in many types of pain conditions but with limited high-quality clinical research.

Concerning treatments for persistent postsurgical pain, Katz et al.^13^ summarize the risk factors for chronic postsurgical pain but with the main emphasis on the implementation of the established Toronto Transitional Pain Service. This approach is unique due to its early identification of significant pain problems and includes a comprehensive care program by a multidisciplinary team in which the preliminary results have demonstrated improvements in pain and pain interference, opioid use, and psychological impairment. Further data, including from other centers with a similar setup, are awaited with major interest. Along the same line, Nowakowski et al.^14^ provide a rationale for a randomized controlled trial on the potential effect of cognitive behavioral therapy to reduce the well-demonstrated significant clinical problem of persistent pain following surgical fixation of extremity fractures. The results of this ambitious randomized controlled trial will again be awaited with major interest.

In summary, and as emphasized in the most recent reviews on chronic postsurgical pain,^1,5^ the many ongoing research activities, including the present special issue articles, support the comprehensive efforts of the Michel DeGroote Institute for Pain Research in better understanding and managing persistent postsurgical pain. These efforts will play an important role in addressing preoperative risk factors, mechanistic studies, and therapeutic approaches in a promising multidisciplinary setting.

## References

[1] Glare P, Aubrey KR, Myles PS. Transition from acute to chronic pain after surgery. Lancet. 2019;393(10180):1537–46. doi:10.1016/S0140-6736(19)30352-6. 30983589

[2] Ji RR, Nackley A, Huh Y, Terrando N, Maixner W. Neuroinflammation and central sensitization in chronic and widespread pain. Anesthesiology. 2018;129(2):343–66. doi:10.1097/ALN.0000000000002130. 29462012 PMC6051899

[3] Kehlet H, Jensen TS, Woolf CJ. Persistent postsurgical pain: risk factors and prevention. Lancet. 2006;367(9522):1618–25. doi:10.1016/S0140-6736(06)68700-X. 16698416

[4] Lavand’homme P, Wu C, Katz J. From acute to chronic postoperative pain. In: Gold MS, Pogatzki-Zahn E, Wallace MS, editors. Pain 2018. Refresher courses, 17th world congress on pain. Washington (DC): IASP Press; 2018. p. 147–57.

[5] Price TJ, Basbaum AI, Bresnahan J, Chambers JF, De Koninck Y, Edwards RR, Ji RR, Katz J, Kavelaars A, Levine JD, et al. Transition to chronic pain: opportunities for novel therapeutics. Nat Rev Neurosci. 2018;19(7):383–84. doi:10.1038/s41583-018-0012-5. 29765159 PMC6237656

[6] Richebe P, Capdevila X, Rivat C. Persistent postsurgical pain: pathophysiology and preventative pharmacologic considerations. Anesthesiology. 2018;129(3):590–607. doi:10.1097/ALN.0000000000002238. 29738328

[7] Quinlan J. Caesarean delivery: bringing more than just a bundle of joy. Can J Pain. 2019;3(1):3–4. doi:10.1080/24740527.2019.1627849. PMC8730553 35005413

[8] Ashoorion A, Sadeghirad B, Wang L, Couban R, Adili A, Guyatt GH, Busse JW. Predictors of persistent post-surgical pain following total knee arthroplasty: a protocol for systematic review and meta-analysis. Can J Pain. 2019;3(1):10–15. doi:10.1080/24740527.2019.1614881. PMC8730639 35005414

[9] Khan JS, Jibb L, Busse J, Gilron I, Choi S, James P, McGillion M, Mackey S, Buckley N, Lee SF, et al. Electronic versus traditional data collection: a multicenter randomized controlled perioperative pain trial. Can J Pain. 2019;3(1):16–25. doi:10.1080/24740527.2019.1587584. PMC8730625 35005415

[10] Ouellette C, Henry S, Turner A, Clyne W, Furze G, Bird M, Sanchez K, EWatt-Watson J, Carroll S, Devereaux PJ, et al. The need for novel strategies to address postoperative pain associated with cardiac surgery: a commentary and introduction to “SMArTVIEW”. Can J Pain. 2019;3(1):26–35. doi:10.1080/24740527.2019.1603076. PMC8730666 35005416

[11] Gilron I, Kehlet H, Pogatzki-Zahn E. Current status and future directions of pain-related outcome measures for post-surgical pain trials. Can J Pain. 2019;3(1):36–43. doi:10.1080/24740527.2019.1583044. PMC8730641 35005417

[12] Linher-Melville K, Singh G. Evaluating the efficay of cannabidiol to manage surgically induced neuropathic pain in a preclinical rat model: are T cells a sexually dimorphic target? Can J Pain. 2019;3(1):44–48. doi:10.1080/24740527.2019.1612235. PMC8730578 35005418

[13] Katz J, Weinrib AZ, Clarke H. Chronic postsurgical pain: from risk factor identification to multidisciplinary management at the toronto general hospital transitional pain service. Can J Pain. 2019;3(1):49–58. doi:10.1080/24740527.2019.1574537. PMC8730596 35005419

[14] Nowakowski ME, McCabe RE, Busse JW. Cognitive behavioural therapy to reduce persistent post-surgical pain following internal fixation of extremity fractures (COPE): rationale for a randomized controlled trial. Can J Pain. 2019;3(1):59–68. doi:10.1080/24740527.2019.1615370. PMC8730643 35005420

